# Supplementary figures and images for: Behavioral factors predict all-cause mortality in female coronary patients and healthy controls over 26 years – a prospective secondary analysis of the Stockholm Female Coronary Risk Study
Source: PLoS One. 2022 Dec 7;17(12):e0277028. doi: 10.1371/journal.pone.0277028 (PMC9728905; doi:10.1371/journal.pone.0277028)

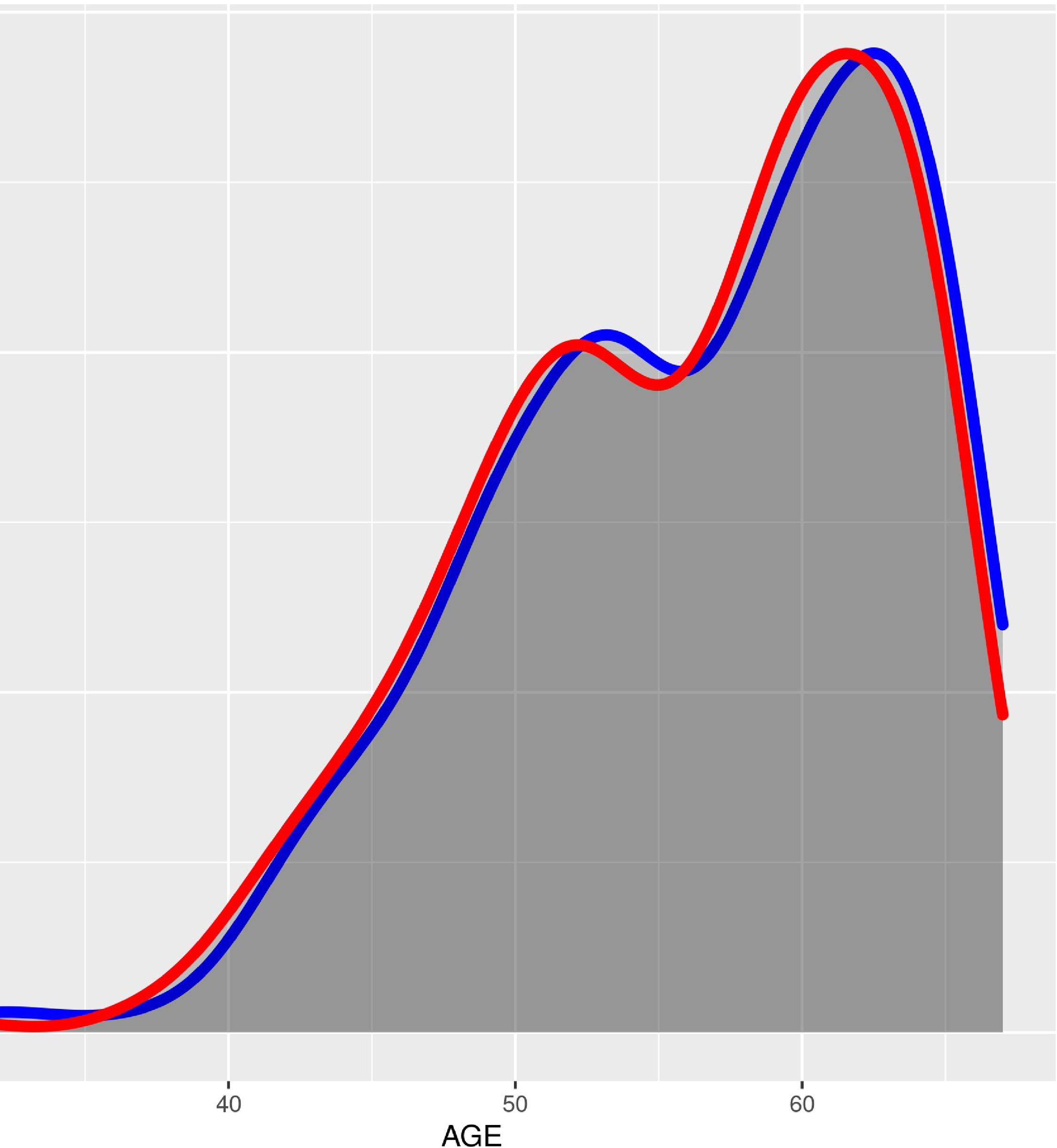

Supplement: S1 Fig — The density plot of the matched ages shows nearly perfect agreement between patients (in red) and controls (in blue). Interesting is the maybe mix of younger and older patients, giving a hint to a heterogeneity in the patients. The curves in Fig 1 have to be interpreted with this age distribution in mind. (TIF) [file pone.0277028.s001.tif]

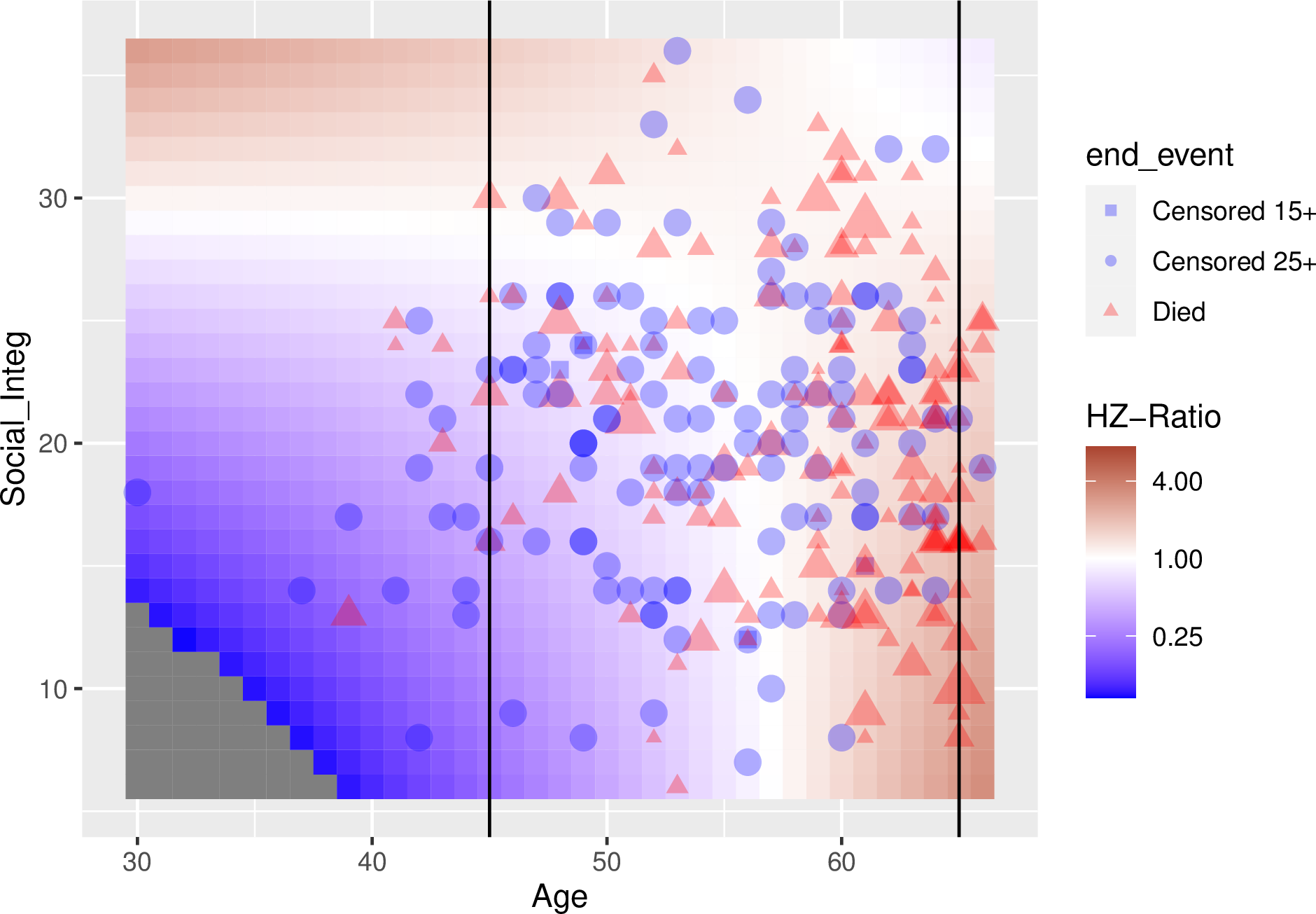

Supplement: S2 Fig — X-axis and the model variable present the age at baseline. The graphic compares the prediction of our model and the mortality data obtained from the study. The two black lines are reference lines, where the hazard ratio (HR) lines (red and light blue) appear as stripes in the background. HR for mortality values >1 is dark red, HR values <1 are shown in blue. The events appear as plots of squares (censored alive at 15-year follow-up), circles (censored alive at 26-year follow-up), or triangles (dead at 26 year-follow-up); larger shapes represent more individuals with the same age and the same social integration score. (TIF) [file pone.0277028.s002.tif]
