# Supplementary material for: Behavioral factors predict all-cause mortality in female coronary patients and healthy controls over 26 years – a prospective secondary analysis of the Stockholm Female Coronary Risk Study
Source: PLoS One. 2022 Dec 7;17(12):e0277028. doi: 10.1371/journal.pone.0277028 (PMC9728905; doi:10.1371/journal.pone.0277028)
Supplement: S1 Table — (PDF) [file pone.0277028.s003.pdf]

S1. Table. Social integration, received stress and behavioral risk factor as predictors of increasing coronary stenosis, recurrent cardiac events, cardiac and all-cause mortality - short-term results within the Female Coronary Risk Study

Authors; publication year	Follow up period (y.); n	Predictors examined	age adjusted Hazard Ratio (95% CI)	Outcome measure
CAD patients				
Orth-Gomér et al 2000 (9)	4.8 y.<br>200	marital stress<br>work stress	3.02 (1.37-.6.65)<br>1.69 (0.72-3.98)	recurrent events
Horsten et al 2000 (30)	5 y.<br>292	depres.symptoms<br>lack of soc. integration	1.9 (1.02-3.6)<br>2.3 (1.2-4.5)	total mortality, card. Mortality, rec. cardiac events
Al-Khalili et al 2002 (3)	5 y.<br>228	index event AMI<br>diabetes mellitus<br>LV-dysfunction<br>HDL cholesterol <1<br>Ser.triglycerides >2	9.1 (3.09-26.99)<br>4.1 (1.68-10.17)<br>3.9 (1.52-10.17)<br>4.0 (1.62-6.12)<br>2.5 (1.06-5.54)	all cause deaths 28 (26%)<br>rec.cardiac events 39 (12%)
Leineweber et al 2003 (27)	5 y.<br>292	poor sleep quality	2.5 (1.2-5.2)	cardiac events
Janszky et al 2004 (23)	9 y.<br>251	SDNN index<br>LF-power<br>HF-power (HRV)	1.56 (1.19-2.05)<br>1.18 (1.07-1.30)<br>1.18 (1.05-1.33)	total mortality<br>cardiac mortality
Wang et al 2006 (16)	3 y<br>131	depression + social isolation	0.18 (-0.11- -0.24)mm	QCA* lumen decreased
Wang et al 2007 (11)	3 y.<br>131	family stress + work stress	0.20 (-0.14- -0,25) mm	QCA* lumen decreased
Zimmermann et al 2013 (21)	3 y.<br>131	vital exhaustion highest quartile	0.21 (-0.15- -0.27)mm	QCA* lumen decreased
Al-Khalili et al 2007 (28)	9 y.<br>273	no exercise testing<br>sedentary lifestyle	4.26 (2.02-8.95)<br>2.94 (1.31-6.62)	total mortality<br>cardiac mortality
Healthy controls				
Leineweber et al 2007 (50)	9 y.<br>264	disturbed sleep<br>work strain	(p=0.07)<br>(p=0.02)	cardiovascular diseases

* Quantitative Coronary Angiography
