# Supplementary material for: Behavioral factors predict all-cause mortality in female coronary patients and healthy controls over 26 years – a prospective secondary analysis of the Stockholm Female Coronary Risk Study
Source: PLoS One. 2022 Dec 7;17(12):e0277028. doi: 10.1371/journal.pone.0277028 (PMC9728905; doi:10.1371/journal.pone.0277028)
Supplement: S2 Table — Kaplan Meier estimates of survival probablility per year of follow up for 286 CAD patients and 299 healthy controls, expressing variability by this coding. (PDF) [file pone.0277028.s004.pdf]

S2 Table: Survival. Kaplan Meier estimates of survival probablility per year of follow up for 286 CAD patients and 299 healthy controls, expressing variability by this coding.

**Time** (years)	**Patients** (survival probability)	**Controls** (survival probability)
**0**	1	1
**1**	0,99301	0,99667
**2**	0,98601	0,99333
**3**	0,98252	0,99
**4**	0,96853	0,99
**5**	0,95804	0,99
**6**	0,92308	0,99
**7**	0,8986	0,98667
**8**	0,88112	0,98
**9**	0,86364	0,96667
**10**	0,83566	0,96333
**11**	0,81469	0,95333
**12**	0,8007	0,93667
**13**	0,79371	0,93333
**14**	0,77972	0,92333
**15**	0,76222	0,92
**16**	0,74115	0,90996
**17**	0,72355	0,89658
**18**	0,70929	0,87651
**19**	0,67365	0,85644
**20**	0,65226	0,83971
**21**	0,60949	0,82298
**22**	0,59167	0,79956
**23**	0,57385	0,76945
**24**	0,52714	0,74938
**25**	0,50187	0,73265
**26**	0,47486	0,67586
**27**	0,44867	0,60855
