# Supplementary material for: Behavioral factors predict all-cause mortality in female coronary patients and healthy controls over 26 years – a prospective secondary analysis of the Stockholm Female Coronary Risk Study
Source: PLoS One. 2022 Dec 7;17(12):e0277028. doi: 10.1371/journal.pone.0277028 (PMC9728905; doi:10.1371/journal.pone.0277028)
Supplement: S3 Table — (PDF) [file pone.0277028.s005.pdf]

S3 Table Test of proportionality in patients after 26 years follow up

	Chi sq	df	p
Social Integration	0.0034	1	0.9536
Age	2.3597	1	0.1245
Phys. activity 24h ECG	0.1092	1	0.7410
Glucose_log	2.5119	1	0.1130
Current smokers	1.8243	1	0.1768
ALT log	2.6749	1	0.1019
Uric acid log	0.6079	1	0.4356
LV dysfunction	2.7543	1	0.0970
AP_log_sd	0.0527	1	0.8185
DHEAS_log	0.1821	1	0.6696
Exercise mod. (WHO)	0.2035	1	0.6519
menopause	5.0327	2	0.0808
Disturbed sleep	0.1173	1	0.7319
Depression	0.0439	1	0.8340
Killip class >1	9.8487	1	0.0017
Work stress	0.0218	1	0.8827
Homemaker	2.4875	1	0.1148
Social integrat. x age	0.3667	1	0.5448
Age x log_AP_sd	0.0304	1	0.8616
GLOBAL	28.9442	20	0.0889
