# Supplementary material for: Behavioral factors predict all-cause mortality in female coronary patients and healthy controls over 26 years – a prospective secondary analysis of the Stockholm Female Coronary Risk Study
Source: PLoS One. 2022 Dec 7;17(12):e0277028. doi: 10.1371/journal.pone.0277028 (PMC9728905; doi:10.1371/journal.pone.0277028)
Supplement: S1 File — (PDF) [file pone.0277028.s006.pdf]

S 1 File. Further descriptions of the study design, baseline examinations and non-significant predictors (in bold) examined in the Stockholm Study of Coronary Risk in Women (1991-1994) at 26-year follow-up.

1. Study design (5)

The Stockholm Female Coronary Risk Study, a population-based case-control study, included all Swedish-speaking female patients 65 years or younger who were admitted to any of the 10 coronary care units in Stockholm for an acute CHD event. The 292 patients were compared with 300 age matched healthy controls by means of a person identification number, which was based on birth date and sex. Control subjects were compared with a random sample of 2500 women of the same age range from the general population of Stockholm (13).

a. Recruitment of CAD patients (3,5)

All patients who needed and sought hospital care for an acute CHD event during this time period could be reached. Patients were included in the study if their hospital records indicated any of the following criteria: (1) definite or suspected MI based on the World Health Organization criteria of typical chest pain, typical enzyme patterns, and diagnostic ECG changes13 (ECG changes were classified by use of the Minnesota code); (2) unstable angina pectoris defined as newly debuted severe angina pectoris that had deteriorated during the last 4 weeks before admission, with an increase in pain intensity and pain duration or with pain at rest or very low physical exertion; or (3) spasmangina, defined as angina pectoris at rest with pathological ST-segment changes on ECG and with normal coronary arteries on acute clinical coronary angiography.

b.Recruitment of healthy controls (14)

For each patient, a healthy woman born on the same day or another day as close as possible who lived in the same hospital catchment area as the patient was chosen. "Healthy" was defined as being free of symptoms of heart disease and without hospitalization for any illness during the prior 5-year period.

2. Methods

A questionnaire on lifestyle and psychosocial factors was mailed to the subjects prior to their visit to the research clinic. Questionnaires were completed at home and brought to the research clinic, where the research nurse reviewed them with the subject to complete missing answers. Anthropometric measures, gynecological interview, blood pressure, and fasting blood samples were all collected and assessed at the research clinic (14).

- *Social examination* (14): **Educational attainment** *was* categorized into 3 groups: (1) low (mandatory or less than high school), corresponding to 9 school years or less; (2) medium (high

school), corresponding to 10 to 13 school years; and (3) high (college/university), corresponding to 14 school years or more. The **occupational status** of patients was estimated according to the Statistics Sweden (SCB): semi\unskilled; skilled; enterpreneuers; clerical&sales work; administrators and executives/ professionals.

- *Self and expert rating of the social situation* (7,34): A condensed version of the Interview Schedule for Social Interaction (ISSI) was used to measure **social support**. The instrument yields two scales, one describing availability of '**social integration**' the more peripheral contacts of social networks. The 'social integration' scale describes both the quantitative characteristics of the extended network and its function. [42]. The other '**attachment**' scale describes the availability of emotional relationships and support, mainly from family and close friends. It consists of six items with a minimum score of zero and a maximum score of six. In a structured interview focusing on all types of family relations—children, spouse, parents, siblings, etc, the **household size** (total number of people living in one's household), **marital status** and **household income** were calculated (14). It was examined to what extent marital stress and the exposure to work-stress was present (12).

- *Further Psychological measurements*: **Coping** with adverse life experiences (42), **measurement of quality-of-life**, **anger scales** (anger symptoms, anger in, anger out), **hostility scale** (MMPI), **perceived stress scale, type A scale** (Framingham), **self esteem scale, extraversion** (social orientation, hedonia; Eysenck), **Life events scale.**

- *Clinical and laboratory examination* (3,5): The severity of **heart failure symptoms** (Killip or NYHA classification) at the time of the index event was abstracted from the medical record and splitted for the regression analysis as follows: no signs of left ventricular decompensation (I) versus basilar rales, tachypnoea (II) or severe left ventricular decompensation/pulmonary oedema (III) or cardiogenic shock (IV). Height (cm) and weight (kg) were measured by a research nurse. **Body mass index (BMI)** was calculated as weight (kg) divided by height (m^2). **Waist-to-hip ratio** was calculated as waist/hip circumference. Method of analysis for **total cholesterol**, **triglycerides, HDL cholesterol** and **LDL cholesterol**, **Lipoprotein A, B and B/A, glucose** and hemostatic factors: **Fibrinogen, factor VII activity, von Willebrand factor** and **PAI-1** have been described previously (1,3,30). **CRP, fructose, uric acid, creatinine, albumin, haptoglobulin, calcium, iron, IBC, AST, ALT, gamma-GT, alkaline phosphatase,** and hormone tests of **cortisol, DHEAS, estradiol, progesterone** were included in the regression. Prescribed medications **ASA, statins, beta-blockers, calcium blockers, HRT, ACE inhibitors, anticoagulants, antidiabetics, insulin,** and **lipid-lowering agents** were also included in the Cox Boost analysis.

- Coronary risk factors (1): **Hypertension**: history of hypertension (according to the physician's diagnosis) or systolic blood pressure above 140 mmHg, or diastolic blood pressure above 90 mmHg measured after 5 min supine rest.

Diabetes mellitus: prescription of anti diabetic therapy in the form of diet or drugs.
Nutrition: **energy intake/per day (Kcal), alcohol consumption,**
Menopausal status was assessed with a gynecological interview by the research nurse. Postmenopausal status was defined as having had no menses for at least 6 months. A complete **history regarding HRT** was also obtained. Women who had begun HRT before menopause were considered menopausal if they were >50 years of age.
-*Selective coronary angiography* (3) was performed according to the Judkins' technique (4). A **stenosis** was considered significant if the vessel diameter was reduced by 50% or more. Significant CAD was considered when significant stenosis was found in at least one epicardial vessel. Ventriculography was performed at the end of each coronary angiography and was visually assessed from two views. Fifty-three of the remaining 292 patients (18%) did not participate in the angiographic part of the study either because of patient refusal or limited laboratory resources at the time of examination. A left ventricular angiography was performed at the end of each angiography. **Left ventricular function** was visually classified as normal or dysfunctional.
-*HRV measurement* (28, 29,38): From the original patient study group (292 patients), 268 patients had Holter monitoring. ECG recordings were excluded if they showed more than 10% nonsinus rhythm (seven patients), or less than 50% of the original ECG recording was available for analysis (eight patients). From the original control study group (300 women), 264 complete 24-hour Holter EKG recordings were available. Recordings were excluded if they showed more than 10% nonsinus rhythm (N = 4), extreme outliers on total power (N = 2), medication for hypertension (N = 3), or less than 50% originalmaterial (N = 6), resulting in 249 recordings available for the analyses. The mean of the SDs of all normal to normal intervals for all 5-min segments of the entire recording (**SDNN index**, in ms) were obtained from the time series of normal RR intervals. Frequency domain parameters were calculated using an auto-regressive method**: high-frequency (HF) power**: 0.15–0.40 Hz, **low-frequency (LF) power**: 0.04–0.15 Hz**, very-low frequency (VLF) power**: 0.0033–0.04 Hz and **total power** (in ms 2) and LF/HF ratio.
