# Supplementary material for: Behavioral factors predict all-cause mortality in female coronary patients and healthy controls over 26 years – a prospective secondary analysis of the Stockholm Female Coronary Risk Study
Source: PLoS One. 2022 Dec 7;17(12):e0277028. doi: 10.1371/journal.pone.0277028 (PMC9728905; doi:10.1371/journal.pone.0277028)
Supplement: S2 File — (PDF) [file pone.0277028.s007.pdf]

S 2 File. Additional statistical description

Variable Selection

After preselecting variables in a two-step approach, we used a machine-learning technique for model building.

There are 191 columns of original input data. First, we choose columns representing general anamnestic information, physiological, disease-related diagnostic measures, clinical chemistry, and social and behavioral indicators, reduced to summary scores, resulting in a set of 80 variables to be considered. We selected the final set of variables after attempts towards model building for two age-defined strata—age below and above median age (56 years) at study entry. Observing different effects and rather unstable estimates in the two strata and recognizing probable under-powering for the two strata, we switched to a model for the entire study group, using the 80 candidates plus age and interactions with age in addition.

Setting up a regression model for survival times is an ambitious task in this situation, as the number of variables **p** is in the same order of magnitude as the number of events **e**. An analysis based on classical Cox regression including all **p** variables is then not possible.

Setting up a regression model

Choosing one of the new machine-learning approaches, the Cox Boost concept **(**39, https://GitHub.com/binderh/CoxBoost**),** which has been used and cited by many authors from various fields of medical research, such as cancer or cardiology (41), we could overcome the **p > = e** dilemma.

The Cox Boost algorithm is an iterative procedure, selecting in every single step the most important variable when added to the last model selected so far. The estimated coefficient of this added candidate is shrunk towards 0 by a penalty factor. This procedure is substantially different from classical

regression. In the statistical literature (41–42), boosted regression is regarded as a good tool for estimating effects within a difficult situation where the total number of explanatory variables is too large for classical approaches.
