# Supplementary material for: Behavioral factors predict all-cause mortality in female coronary patients and healthy controls over 26 years – a prospective secondary analysis of the Stockholm Female Coronary Risk Study
Source: PLoS One. 2022 Dec 7;17(12):e0277028. doi: 10.1371/journal.pone.0277028 (PMC9728905; doi:10.1371/journal.pone.0277028)
Supplement: S3 File — (PDF) [file pone.0277028.s008.pdf]

S3 File. Details of the statistical analysis

Using a machine-learning based method like Cox Boost bears two sources for misinterpretation of results: overfitting by too complex modeling and unreliable estimates due to small subgroups with an extreme pattern of covariates that can lead to high leverage points with substantially influence on parameter estimates. We handled these challenges in a pragmatic way: (i) we adapted the number of boosting steps by choosing a high penalty and limiting the number of variables selected in advance - this avoids overfitting - and (ii) in case of very small subgroups by rare values of a categorical covariate, we recoded these values into fewer categories avoiding small subgroups. (iii) we augmented the classical final cox-regression model providing the results presented by interactions with age for variables where age-specific effects were detected in the selection process. These steps were not carried out through an automated process, but in a pragmatic way determined by the statisticians involved.
